# Rye Bran as a Component in the Diets of Lactating Sows—Effects on Sow and Piglet Performance

**DOI:** 10.3390/ani14030380

**Published:** 2024-01-24

**Authors:** Christian Homann, Volker Wilke, Isabell Eckey, Bussarakam Chuppava, Martin Kaltschmitt, Andreas Zimmermann, Christian Visscher

**Affiliations:** 1 Institute for Animal Nutrition, University of Veterinary Medicine, Foundation, 30559 Hannover, Germany; christian.homann@tiho-hannover.de (C.H.); sekretariat-tierernaehrung@tiho-hannover.de (I.E.); christian.visscher@tiho-hannover.de (C.V.); 2 Institute of Environmental Technology and Energy Economics, Hamburg University of Technology, 21073 Hamburg, Germany; kaltschmitt@tuhh.de (M.K.); andreas.zimmermann@tuhh.de (A.Z.)

**Keywords:** rye, rye bran, pigs, sows, piglets, nutrition, by-products, performance

## Abstract

**Simple Summary:**

The use of by-products such as rye bran in sow diets is interesting in terms of functional ingredients, cost, and sustainability. Rye bran has many positive properties for the intestinal health of sows, so its use in their diet is appropriate. In this study, the effects of rye bran in a compound feed for lactating sows were investigated with regard to performance in terms of loss of body weight and backfat thickness in the sow as well as weight gains in piglets. There was no significant effect of rye bran on the performance of sows concerning the above parameters. The daily weight gains of the piglets also showed no differences.

**Abstract:**

From a cost and sustainability perspective, the use of by-products such as rye bran in sow diets is of particular interest. Rye bran has valuable ingredients that have potential benefits for the gut health of sows. The aim of this study was to investigate the effects of including 15% rye bran in the sows’ feed on the performance of sows and piglets. The feeding started one week before the farrowing date and ended at weaning. Performance was evaluated by measuring sow (n = 175) and piglet body weight (n = 1372) and sows’ backfat thickness (n = 80). These data were additionally used to calculate the colostrum intake of the suckling piglets and the sows’ milk production. It was found that there were no differences in the performance parameters between the experimental and control groups. However, this study showed that the piglets with light birth weight (LBW (<1000 g)) and medium birth weight (MBW (1000–1500 g) consumed more colostrum when the sows were fed rye bran (LBW: C/R 203.0 ± 39.2 g/214.3 ± 35.9 g; MBW: 291.3 ± 39.0 g/298.5 ± 36.4 g). It can be concluded that including 15% rye bran in the feed of lactating sows has no obvious negative effects on the performance of sows and piglets. Further studies are needed to evaluate the possible positive effects of rye bran.

## 1. Introduction

Rye (*Secale cereale*) is a typical bread and feed grain in Northern Europe [1]. In terms of cultivation, rye has many advantages; above all, it has high drought resistance as well as high nutrient efficiency and is, thus, highly adaptable to nutrient-poor soils [2]. This makes hybrid rye, which has significantly increased in yield through breeding efforts [3], attractive for cultivation in the future, especially regarding climate change and fertilizer restrictions. Compared to other common feed grains for pigs, such as barley, wheat and triticale, rye has a similar nutritional profile [4]. In the past, corn, wheat and triticale were more attractive alternatives for various reasons, such as their valuable ingredients without the risk of ergot contamination [1]. Nonetheless, rye has several advantages, such as having a lower *Fusarium* toxin load compared to wheat, which is mainly used, but the risk of ergot contamination is higher [5]. Ergot alkaloids, which arise from *Claviceps* spp. fungus, can be harmful to organisms [6]. Ergot alkaloid poisoning in pigs manifests as vasoconstriction, which can lead to reproductive disorders, abortions, agalactia and growth delays [7,8]. For this reason, worldwide cultivation has been steadily reduced in recent decades [9]. Breeding efforts have made it possible to reduce the ergot load in rye plants [1]. This has been achieved primarily through increased pollen dispersal in rye hybrids, which results in a lower ergot load [10], allowing rye to be used in animal feed without the risk of ergot contamination [1].

Therefore, closed local feed cycles are becoming increasingly important. The use of by-products offers the possibility of adding low-cost products to animal feed in a resource-efficient way [11]. In this regard, the food industry offers high-quality components [12,13]. The use of by-products is also important in terms of sustainability (circularity) [13]. A by-product of rye processing is rye bran, which is produced during the production process of rye flour. Rye flour mainly consists of the outer part of the kernel and the remaining endosperm [14]. In general, bran has a high content of dietary fiber, vitamins, minerals and other bioactive substances [14]. The nutrient composition makes it attractive as a feed component [1,15]. The dietary-positive effects of rye are due to the high content of fermentable dietary fiber, in particular, non-starch polysaccharides (NSPs) [4]. When comparing the chemical structures of wheat bran with those of rye bran, significantly higher proportions of fructans and pentosans, which proportionally form an important part of NSPs, are found in rye bran. Microorganisms are able to metabolize these in the host’s large intestine. Since rye bran contains more NSPs than wheat bran [14], more metabolic processes may take place in the large intestine of the pig when receiving rye bran. During this process, volatile fatty acids are formed, including the short-chain fatty acid (SCFA) butyrate [16,17]. In the colon, butyrate supports epithelial barrier function via enterocyte nutrition [18]. Other studies have shown that butyrate reduces the invasion of intestinal epithelial cells by *Salmonella enterica* [19].

Previous research results have shown that sows fed lactating diets containing 35% hybrid rye performed as well as sows fed control diets based on barley, wheat and soybean meal [20]. However, so far, there are no data on how rye bran as a component in a feed concept affects sow and suckling piglet performance. The performance parameters in sow management comprise data such as the number of live-born piglets, weaned piglets and daily weight gains. Important aspects of lactation performance include ensuring the optimal condition of the sow at the beginning of lactation and that the loss of body reserves remains within physiological limits [21,22]. Body weight (BW) and backfat thickness (BFT) one week before farrowing and at the end of lactation serve as measurement parameters for the development of body condition during lactation [22,23]. Studies showed that sows that lost too much weight during lactation had piglets with less consistent birth weight following lactation [24]. Optimizing feeding with rye bran as a component could, via improved intestinal health, reduce instances of decreased body condition and, thus, positively influence lactation and, consequently, the piglets. In addition, diseases such as Postpartum Dysgalactia Syndrome (PPDS) and Mastitis may be prevented through the beneficial effects of rye bran on intestinal transit. In particular, rye bran offers the possibility of replacing other feed grains, providing an economic incentive in addition to potential animal health effects. 

Our hypothesis was that the addition of rye bran to the diet of lactating sows improves the performance of sows and piglets. The main objective was to investigate the effect on sow weight and backfat thickness against a control group before and after nursing. In addition, the effects of rye bran on piglet weight were evaluated and compared with a control group. The colostrum intake and the amount of milk produced by the sow were then calculated from the data obtained. The aim was to clarify whether the addition of rye bran is beneficial to sow and piglet performance within the parameters described. 

## 2. Materials and Methods

### 2.1. Ethical Statement

The animal experiment was approved by the Ethics Committee for Animal Experiments of LAVES (Niedersächsisches Landesamt für Verbraucherschutz (Lower Saxony State Office for Consumer Protection): file number 33.8-42502-05-20A557). The experiment was part of the Rye-SaFe project (2813IP026), which is funded by the German Federal Ministry of Food and Agriculture.

### 2.2. The Farm and Animals

The experiment was conducted from March to December 2022 in a piglet breeding herd in Northern Germany. The farmer’s participation in the study was voluntary. The farm kept about 1000 breeding sows of BHZP Viktoria genetics in two herds of 500 animals each. The sows were inseminated with Danish boars (DD+, BHZP GmbH, Dahlenberg-Ellringen, Germany). The sow herd was restocked with purchased gilts, which were subsequently integrated into the herd after a quarantine period. Animals were randomly selected for the experiment. A total of 80 peripartum sows (of a total of 200 in this stable) in the two compartments with their associated 1373 piglets were selected for the entire experiment. The study was conducted in four runs, with 10 sows per feeding group (control or experimental). There was a random but representative mix of parities in each group (average parity control (C): 4.34 ± 2.04; experimental (E): 4.29 ± 1.83). Parturition was induced by injection of cloprostenolum (Veyx-Pharma GmbH, Schwarzenborn, Germany) so that farrowing occurred at the calculated time. The sows, thus, had a gestation period of 115 days. The suckling period of the piglets was three weeks, after which all piglets were weaned on day 21. During the suckling period, the piglets were kept together with the sow in a farrowing pen according to currently valid standard sizes (6.5 m^2^ ground area per farrowing pen). It was equipped with a heating plate and, if required, additional infrared lamps. After birth, the piglets remained with the sow for 24 h, and, if necessary, cross-fostering of piglets was performed within the same feeding group. After three weeks, the piglets were weaned and moved to the rearing unit.

### 2.3. Diets

The farm-specific purchased compound feed for lactating sows containing wheat bran was used as the control feed, while the experimental feed contained rye bran instead. The commercial control feed (HL HAMBURGER LEISTUNGSFUTTER GmbH, Hamburg, Germany) contained wheat, barley and soybean extraction meal as main components. It also contained wheat bran as fiber component. On this basis, an isoenergetic (ME) experimental diet with the same crude protein content (HL HAMBURGER LEISTUNGSFUTTER GmbH, Hamburg, Germany) with 15% rye bran was calculated, which no longer contained wheat bran. In runs 3 + 4, and the feed had to be changed because corn was not available as a component in the feed mill. Since the feed manufacturer could not predict when they would be able to deliver the original diets, we had to design two new diets that did not contain corn. The specifications (isoenergetic, same protein content, 15% rye bran in the experimental diet) were identical to the first two runs. Two different control diets were used during the trial period. An experimental diet with 15% rye bran was adjusted to both of these diets. To achieve the same energy and protein content, the composition of the feed had to be adjusted and so did the proportions of the other feed components. The composition of the sows’ control and experimental diets is shown in Table 1.

The calculated nutritional values of the control and experimental diets are summarized in Table 2. 

**Figure 1:**
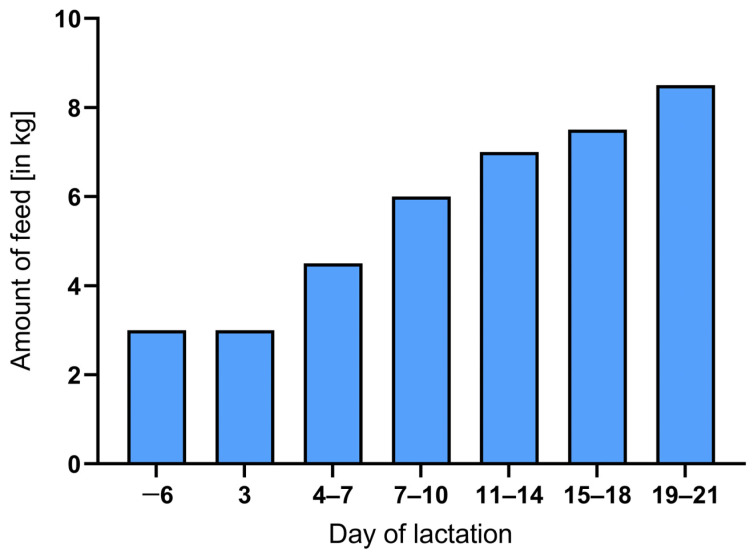
Feed allocation increase (in kg) per sow during lactation.

The meal-based compound feeds were dry fed to the animals through individual volumetric feeders. The amount of feed for the sows was continuously increased during lactation, as shown in Figure 1.

From day 7 after birth, the piglets were additionally fed with a pelleted pre-starter diet (Una-Hakra, Immuno G, Una-Hakra, Hanseatische Kraftfuttergesellschaft mbH, Hamburg, Germany) via piglet trays.

During the study, the control and experimental feeds were analyzed regularly. Two feed samples (1.5 kg) were collected per run, which were then pooled. An aliquot of each sample was taken during each procedure to obtain a representative sample. From the representative 1.5 kg sample, 100 g was subdivided for analysis by using a sample divider. This sample then served as the basis for the analysis. Furthermore, the diets were analyzed for crude nutrients using Weender analysis in accordance with the official methods of the Association of Agricultural Research and Testing Institutes (Verband Landwirtschaftlicher Untersuchungs- und Forschungsanstalten (VDLUFA)) [25]. In addition, the starch and mineral content in the diets were determined. The starch content was used to calculate the energy content (ME) of the feed using the results of the Weender analysis. Mycotoxin analysis was performed using high-performance liquid chromatography (HPLC) to detect the relevant toxins deoxynivalenol (DON), zearalenone (ZEA) and ergotamine. Ergotamine, as one of 12 ergot alkaloids, served as an indicator for the ergot alkaloids. The risk of contamination with mycotoxins or undesirable ingredients was, thus, detected. The analysis of fructane and pentosane content was performed by an external laboratory (Institute of Environmental Technology and Energy Economics, Hamburg University of Technology, Hamburg, Germany) [26].

### 2.4. Experimental Design

The experimental phase lasted 28 days, starting with the sows’ entry into the farrowing pen one week before farrowing, to evaluate the entire lactation period. The respective run ended with the weaning of the piglets and the removal of the sows from the pen. In each of the four runs, the performance of the two feeding groups in the peripartum period was studied in parallel to exclude possible environmental effects such as different weather conditions.

#### 2.4.1. Sows

Two farrowing groups from April to May 2022 and two farrowing groups from September to December 2022 were observed. The study was interrupted during the summer to minimize reduced feed intake during high temperatures. The sows were moved to the farrowing unit one week before the calculated farrowing date, which corresponds to the normal work routine of the farmer. Ten sows per run and feeding group were randomly chosen in the experimental and control compartment. The two compartments were separate from each other but almost identical in construction. To exclude housing conditions as a factor, the compartments of experimental (E) and control groups (C) were switched after each run. Feeding the lactation diet started seven days before the farrowing date. At this time, sows were moved into the farrowing barn. After 21 days of suckling, the piglets were weaned, and the sows were moved to the mating center.

#### 2.4.2. Piglets 

**Figure 2:**
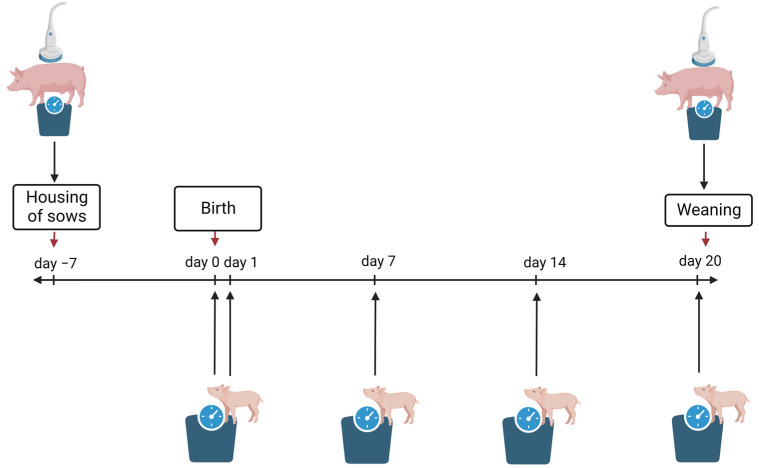
Investigation scheme for the peripartal period and timepoints (created with BioRender.com, accessed on 11 November 2022).

Piglets n(C/E = 713/690) from 80 L n(C/E = 40/40) were examined in four runs. In each of the four runs, data were collected from 10 randomly chosen sows with their associated piglets. The investigastion scheme can be seen in Figure 2. 

### 2.5. Performance Measurement

#### 2.5.1. Sows

**Figure 3:**
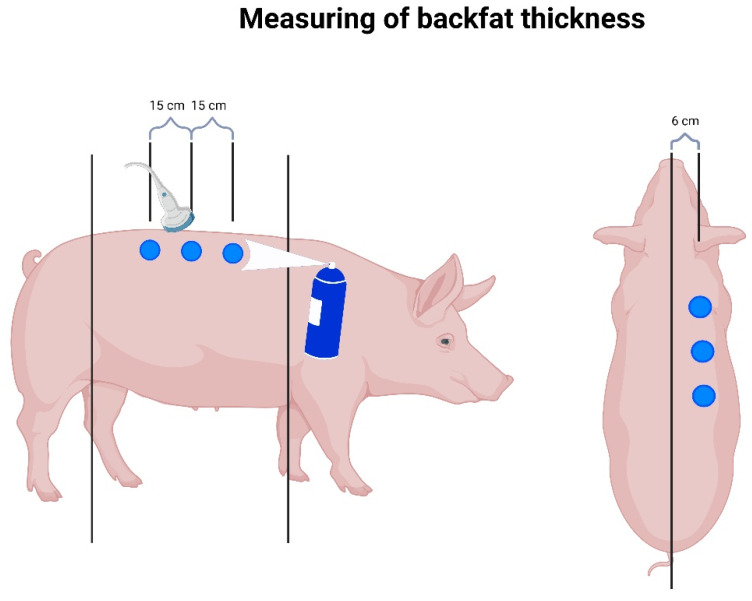
Measuring backfat thickness using Lean-Meater^®^ (created with BioRender.com, accessed on 11 November 2022).

Body weight (BW) and backfat thickness (BFT) of sows were measured seven days before farrowing and at weaning (Figure 2). Since all sows in the compartments were weighed at stall-in and stall-out, the data of all sows in the compartment were considered for sow body weight loss. One compartment had 26 farrowing pens and the other 20. In four runs, each feeding group used each compartment twice, so that comparability was established. The backfat thickness of 10 sows per run per experimental and control group was measured with the Lean-Meater^®^ (Renco Corporation, Golden Valley, MN, USA) using ultrasound sonography, as described by Müller et al. [27]. For this purpose, a stencil was made, which had three pointed cutouts in order to achieve identical distances of the measuring points in all animals. This template was placed in the middle of the animals’ backs so that three measuring points could be marked on the right side of the back [27] (Figure 3). These measurement points were located 6 cm paramedian to the spine [27]. Starting from the middle point, halfway between the scapula and the knee of the sow, every 15 cm cranial and caudal of this middle point, the other two measurement points were marked [27]. An animal paint spray was used for marking so that the points were still visible during the second measurement. The measurement of the standing sows was taken with the help of ultrasonic gel by one person. Backfat thickness was calculated as the average value of the three measurement points. The body weights of all sows in the control and experimental compartments (n = 175) were measured on sow scales (EAG80-E, T.E.L.L. GmbH, Vreden, Germany).

##### Milk Production

According to Flachowsky et al. [28], milk production of sows during lactation was estimated at 4.1 L per 1 kg body gain of suckling piglets. However, since the sows farrowed on different days and, thus, did not lactate for the same length of time (21 or 22 days) until weaning, an average of the milk quantity per lactation day was determined for better comparability. 

#### 2.5.2. Piglets

Piglet weights were determined by using a pair of scales (EOB 60K20, KERN & SOHN GmbH, Balingen-Frommern, Germany). The piglets were individually numbered at birth to determine the exact weight gain per piglet at birth and after 24 h. In total, piglets were weighed on days 0, 1, 7, 14 and 20 to determine the daily weight gain from birth to weaning calculated from these values (Figure 2). In accordance with Theil et al. [29], the colostrum intake of each piglet in the first 24 h after birth was estimated:

CI = −106 + 2.26 WG + 200 BWB + 0.111 D − 1414 WG/D + 0.0182 WG/BWB(1)

Cl (g) = Colostrum WG (g) = Weight gain BWB (kg) = Bodyweight birth.
D (min) = Suckling duration in hours from birth to 24 h after birth of first piglet.
From the sum of these, the colostrum intake of all piglets in the litter was calculated.

In order to also classify the colostrum intake in the different weight groups of the piglets, the animals were divided into three categories. The light piglets (C/E n = 170/176) weighed a maximum of 1000 g, because below this weight, the mortality before weaning increases significantly [30,31]. The cutoff between medium (1000–1500 g) (C/E n = 367/377) and heavy (>1500 g) (C/E n = 160/122) piglets was chosen because, above 1500 g, birth weight has no influence on piglet mortality [30,31].

### 2.6. Statistical Analysis

The SAS Enterprise Guide (version 7.1, SAS Institute Inc., Cary, NC, USA) was used for the statistical evaluation. The performance parameters of sows and piglets (body weight and backfat thickness) were evaluated at individual animal level. The body weights and backfat thicknesses of the sows were calculated with the differences between one week before farrowing and weaning in order to achieve a better comparability of the data. A test for normal distribution was performed using distribution analysis by means of the Shapiro–Wilk test for analytical evaluation. To show differences in the above parameters between the feeding groups, a *t*-test was performed for the normally distributed data with the control and the experimental groups as independent factors. For the non-normally distributed data, the Wilcoxon rank sum test was used. A significance level of *p* < 0.05 was considered significant for differences. Piglet weights were analyzed using a mixed model, a two-factorial analysis of covariance with repeated measurement (days) and runs as a covariate with consideration of all interactions. To maintain experiment-wise error, the alpha of 0.05 was adjusted according to Bonferroni to alpha = 0.01 for five comparisons.

## 3. Results

### 3.1. Diets 

To test the suitability of rye bran as a feed component, rye bran was first compared with rye as a grain. This ensured that the rye bran contained the valuable ingredients of rye. The valuable NSPs were also found in the rye bran (Table 3).

The experimental and control diets used had an equal crude protein content but contained different compositions of NSPs (Table 4 and Table 5). The ergotamine level, as an indicator of the ergot alkaloids, in the diets was below 10 µg/kg, which is the detectable limit. However, our analyzed feed was far below the limit values for mycotoxins in feed for sows stated in the EU recommendation (guideline values of the EU (Commission Recommendation No. (2006/576/EC) of 17 August 2006) for DON with 0.9 mg/kg and ZEA with 0.25 mg/kg, respectively.

When comparing the raw material used (wheat bran and rye bran; Table 5), it was noticeable that 144% more fructans were present in the rye bran. Then, looking at the total pentosans, higher amounts were found in the rye bran. On comparing the fructan and pentosan content of the control and experimental diets, it was noticeable that the experimental diet contained higher amounts of fructans. The total pentosan content was similar in both diets.

### 3.2. Sow Performance

No differences were found in the weight of the sows at housing (one week before the calculated farrowing date) and at weaning (four weeks later). A comparison of weight loss during lactation also showed no significant differences.

When looking at backfat thickness, it is noticeable that the sows in the experimental group had less backfat thickness at weaning than those in the control group (13.0 ± 2.38 vs. 11.8 ± 2.58; *p*-value 0.03). However, a comparison of the decrease in backfat thickness during lactation showed that neither group lost significantly more BFT (Table 6).

### 3.3. Piglet Performance 

The piglets in the control group and the experimental groups were not different at any of the measured times (Table 7).

The piglets’ weight gains, therefore, did not differ at any time.

In addition, the numbers of piglets born alive and the live born, stillborn, mummified, abnormalities and number of weaned piglets were not significantly different (Table 8).

When comparing the estimated average colostrum intake per suckling piglet, the light (<1000 g birthweight) and medium weight (1000–1500 g birthweight) suckling piglets in the experimental group consumed significantly more colostrum (Table 9).

When comparing the estimated milk production of sows in lactation, the control and experimental groups produced approximately the same amount of milk. There was no difference in the estimates of average daily milk production per sow when the data were compared (Table 10).

## 4. Discussion

Interest in rye as a feed component has steadily increased in recent years; this is not only because of its sustainability [32]. At a time when grain prices are continuing to rise and summers are increasingly characterized by drought, rye is a viable alternative to common cereals, such as wheat, barley and corn [33,34]. Ergot contamination remains a real problem with many types of rye [35]. To ensure safe use in breeding, care should be taken to grow and feed rye that is specifically low in ergot. Farmers who mix their own feed and use their own rye should be alert to the presence of ergot, which can lead to reduced milk yield and abortions [8]. Breeding efforts have greatly reduced the risk of ergot contamination, making it possible to use larger amounts of certain rye varieties (or its by-products) in breeding animals [1]. The analyses did not show ergotamine levels above 10 µg/kg, so the results can be discussed and interpreted without the influence of mycotoxins. Due to the possibility of using pure rye, the use of rye bran as a food by-product is also obvious. 

### 4.1. Diets

Since bran makes up about 10–15% of the grain weight, a relatively large amount of bran is left over during milling [36]. The production of rye meal, thus, produces large amounts of bran that can be mixed into pig feed. Rye bran as a by-product has the positive functional properties of rye and is also a high-fiber feed component. The literature has so far provided little information on the feeding of rye bran, so that in the following, the data of this study will be discussed alongside those of rye and other fiber-rich feed components. McGhee et al. [37] showed that using 25% and 50% rye instead of corn in the compound feed resulted in improved lactation performance, and 75% rye instead of corn resulted in the same performance as the control group. Other previous studies demonstrated the positive effects of dietary fiber components and rye in pig production [38,39]. A comparison of hybrid rye with rye bran as the raw material shows that with increased crude protein, crude fat and crude ash, more crude fiber is also present (Table 4). Rye bran as a feed component is rich in dietary fiber and, thus, combines the possibility of using the positive effects of a high-fiber component with the positive effects of the functional ingredients of rye. Fructans and pentosans as part of the dietary fiber are increased in rye bran [40]. As shown in Table 5, the amount of fructans was higher in the experimental diet than in the control diet. However, when total pentosans are considered, it is noticeable that nearly the same total pentosans were found in the diet, despite the increased proportion of rye bran (9% wheat bran to 15% rye bran in feed). Due to the high amount of total pentosans in the pure rye bran, a higher content of these in the total diet was expected. One reason for this may be that barley and flaxseed are also feed ingredients rich in pentosans [41]. Additionally, the composition of the feed, which had to be adjusted to achieve the same energy and protein content, may have an influence on the NSP content, because different proportions of other feed components (e.g., barley) were also used in the experimental diet. It must also be taken into account that no sugar beet pulp molasses was used in the control feed in the first two runs, while 2.0–2.5% sugar beet pulp molasses was used in all other feeds. Sugar beet pulp is also rich in soluble fiber, which may have had some influence on the differences on the NSP level of the feeds [42]. However, it is not assumed that the lack of 2% sugar beet pulp molasses in the first two runs had such a large influence on the performance parameters.

The results showed no clear effect overall, so perhaps too little rye bran was used in the diet to have a clear effect. Rye bran is a high-fiber component but relatively low in energy. The period around farrowing and lactation is one of the most energy-demanding times for sows [43]. Therefore, the amount of rye bran used around parturition is limited. In other areas of pig production, where a lower energy density of the diet is required (e.g., extensive fattening, or sows in early pregnancy), a higher percentage of rye bran could be used. A targeted use of rye bran in the first transition period, i.e., from before birth to after birth, offers many possibilities. On the one hand, rye bran could serve as a prophylaxis against “Mastitis, metritis and agalactia” (MMA) or “Postpartum Dysgalactia Syndrome” (PPDS) via the fiber and, on the other hand, the rye would contribute to healthy intestinal flora in the sow, which would also have an effect on the piglets. A diet with a higher proportion of rye bran is also conceivable, since there is no energy deficit from milk production. Thus, rye bran could possibly be well used in a two-phase feeding concept around birth.

### 4.2. Performance of Sows 

To maximize the number of live births and future reproductive success in sows, sows’ backfat thickness and body condition in particular have been found to be essential [44]. There are many studies that have looked at the performance of pigs fed a rye-rich diet. Chuppava et al. [45] and Wilke [17] found no negative effects on growth performance when young pigs were fed diets containing a high percentage (69%) of rye. Chuppava et al. [45] showed in a *Salmonella* infection trial that feeding young pigs a compound feed containing 69% rye had no negative effect on performance compared to pigs fed 69% wheat. Furthermore, up to 69% rye in the diet compared to wheat did not negatively affect the performance of young pigs [17]. Other previous studies showed similar results on the effects of feeding rye on pig performance [37,46].

#### 4.2.1. Body Weight and Backfat Thickness of Sows 

Adequate feed intake is essential to provide adequate amounts of energy for sows, especially when sows are nursing large litters [47]. Maintenance and energy requirements are often not covered by feed consumption, which leads to a physiological mobilization of body reserves [47]. Weight loss due to the birth of piglets, placenta, amniotic fluid, etc., is an influencing factor that can vary greatly between sows. Thus, the comparison of backfat thicknesses is a better comparison parameter in this context [48]. In combination, both can be used to describe the energy loss of sows during lactation. 

High levels of dietary fiber can impair nutrient utilization, feed intake and, consequently, energy supply [49]. It was also shown that sows receiving a lower daily energy intake during lactation lose more weight and backfat than those with a higher intake [50]. As a result, the use of more fiber-rich components is uncommon in the diets of lactating sows [51]. In the present study, for these reasons, only 15% rye bran was mixed into the feed.

According to Farmer et al. [23], a moderate body condition (either too low (<15 mm) or too high (>26 mm backfat thickness)) is recommended. Hoving et al. [52] describes that high (>13.8%) body weight loss of sows during lactation affects embryo survival in the following pregnancy. Thus, minimizing sows’ loss of body weight and backfat thickness during lactation is important for reproductive performance in the following pregnancy. In the study by Weng [53], 20% wheat bran, soybean hulls and rice hulls were compared as dietary fiber components in diets for sows where body weight and backfat thickness were evaluated. In that study, no differences in performance were found between the different fiber components in the proportions used. In this study, the sows in the experimental group had less backfat at weaning than the sows in the control group. However, as the sows used were very different (weight, parity, etc.), there was a high standard deviation in backfat thickness, even when the sows were housed. For better comparability, the loss of backfat thickness was determined for each individual animal and compared between the feeding groups. This loss of backfat thickness is the only way to establish comparability between the groups, so that it can be regarded as a relevant parameter. There was no significant difference between the feeding groups. Quesnel et al. [38] and Li et al. [54] also showed that the loss in backfat thickness did not differ, even when high-fiber feed was fed during gestation and lactation. Those results are consistent with the results of this study regarding the loss of backfat thickness.

The feeding of rye bran, as a by-product of rye, is also interesting in other regards. Dietary fiber components in rye can provide energy through fermentation in the hindgut [38]. NSPs as an energy source can, thereby, promote higher butyrate production and improve intestinal health [19]. McGhee et al. [37] showed that feeding high levels of rye had no effect on the body weight loss of sows. In other studies, similar results are also described in younger pigs [17,45]. These results are confirmed by our own data.

Body weight as a performance parameter is greatly influenced by other feed components and nutritional values such as energy density [50]. Indeed, it is described that high-energy diets in lactation lead to a reduced loss of body weight and backfat thickness [55]. Since the energy content in the control and experimental diets was identical, no significant differences were expected. The fact that the body weight and backfat thickness of the sows did not differ between the feeding groups during the study shows that the performance of the sows was not negatively affected by the inclusion of rye bran in the diet. To determine a possible difference due to rye bran, further experiments would have to be conducted using different amounts of rye bran at the same energy content in the peripartum, similar to those used by Wilke [17] in young pigs. A trial with different levels of rye bran up to the maximum utilization of rye bran in the diet of lactating sows would be an approach to better illustrate the possible effects of rye bran.

#### 4.2.2. Milk Production of Sows

Noblet et al. [43] described that there is a close correlation between piglet gain and the amount of milk the sow produces. The literature reports that sows had to produce an average of 4.1 L of milk to produce one kilogram of body weight gain in piglets [28]. Nevertheless, the milk yield of sows can vary considerably and depends on the protein and energy intake in the sows’ feed [56]. Due to the possible positive effect of NSPs in rye bran and the energy provided by the fermentation, it can be assumed that the sows have a more constant energy supply [38]. This refers mainly to the fact that there is less fluctuation in the insulin response, even with few meals a day. This effect could not be demonstrated in our study, partly because the estimated values did not represent the exact milk production of the sows. Further studies are needed to show a possible effect.

### 4.3. Piglet Performance

A high weaning weight of the suckling piglets is essential for the animals’ long-term health but also for the profitability of the farm [57]. Many factors play a role in this context, such as age of the sow, conformation, management and of course feeding [57].

Colostrum intake is the most important factor in piglet survival, providing energy and immune protection, and has potential long-term effects on piglet growth and immunity [58]. According to the latest research, 200 g of colostrum per piglet in the first 24 h after birth was considered the minimum to significantly reduce mortality before weaning, to ensure passive immunity and to allow for weight gain [59]. To achieve good health and growth before and after weaning, a consumption of 250 g is recommended [59]. A total of 11.3% of the control group and 8.87% of the experimental group of suckling piglets consumed less than 200 g of colostrum, while 70.3% of the control group and 75.6% of the experimental group of suckling piglets had an estimated colostrum intake of more than 250 g of colostrum. Devillers et al. [58] described that an adequate colostrum intake can have long-term effects on piglet growth from three weeks of age until after weaning. The classification of the suckling piglets in our study into the three weight categories was made in accordance with Zotti et al. [30] and Vande Pol et al. [31]. A comparison of the weights of the piglets used in the former study shows that they had an average birth weight of 1.36 ± 0.21 kg [30]. The piglets in our study had a mean birth weight of only 1.23 kg ± 0.32 kg, which may require some downward adjustment of weight categories. As the results would be little affected by these adjustments, the categories described by Zotti et al. [30] and Vande Pol et al. [31] were retained. When looking at the average colostrum intake of the suckling piglets, it is noticeable that the light piglets (<1000 g) and medium piglets (1000–1500 g) in the experimental group had a significantly higher colostrum intake than the piglets of the same category from the control group. For this reason, the above-mentioned transitional use of rye bran in the first phase of sows around parturition would also be conceivable. The lighter piglets, in particular, could potentially benefit from this measure.

The nutrition of the pregnant sow up to birth is of great importance for birth, piglet health and piglet productivity and is widely described in the literature [60,61]. Many different studies have previously evaluated the influence of a high-fiber diet in late pregnant or lactating sows on piglet performance. A study by Loisel et al. [62] showed that feeding a high-fiber diet before birth had no effect on piglet weight gain. In addition, the literature shows either positive effects of fiber [63,64] or no effects at all [65]. When comparing piglet weights, no differences were found between feeding groups. Therefore, no effect of rye bran can be described. Since the present study was primarily concerned with the nutrition of lactating sows and not with pregnant sows, the following factors had to be taken into account when producing the diets. As already mentioned, high levels of dietary fiber in the feed may affect nutrient utilization, feed intake and, consequently, energy supply of the sows [49]. As our study was conducted as a field trial on a conventional farm, higher concentrations of rye bran were not used in order to avoid the risk of a significant loss of performance. Performance losses were not observed in the sows, so that no differences in piglet performance were to be expected.

In order to see a positive effect on the intestinal health of sows and piglets under the influence of rye bran, further studies under experimental conditions must work with higher proportions and evaluate parameters other than pure performance. It can be assumed that even if a higher proportion of rye bran is used in practice, no improvement in sow and piglet performance can be expected. Particularly, the mentioned use in a two-phase feeding around farrowing is an approach that needs further research. The amounts of rye bran in a compound feed for lactating sows are already exhausted for the reasons mentioned above. It is also interesting to investigate the influence on colostrum quality and composition with a rye-bran-rich diet. The results of this study show no differences in performance when rye bran is included in the diet of lactating sows, but more research is needed to fully recommend the use of rye bran.

## 5. Conclusions

No differences in body weight loss of the sows and loss of backfat thickness were observed when feeding 15% rye bran in the diet during lactation. The litter size and farrowing rate were not affected, nor was milk yield. This study showed that the light piglets (<1000 g birth weight) and the medium piglets (1000–1500 g birth weight) consumed more colostrum when the sows were fed rye bran. No ergotamine levels above the reference value were detected in the feed, which serve as indicators of possible ergot contamination. Despite the increased NSP levels in the experimental diets, no clear effect of the amount of rye bran on performance could be demonstrated. This study, therefore, shows that rye bran, as a by-product of the food industry, may have the potential to be used in animal feed. Further studies are needed to clearly demonstrate the effects of rye bran on sow and piglet performance. In particular, the possible temporary use in sow feeding around farrowing is of future interest due to the proven effect on sows and piglets.

## Figures and Tables

**Table 1:** Composition of the control as well as the experimental diets for lactating sows.

Ingredients	Control	Experimental	Control	Experimental
(% uS)	1 + 2	1 + 2	3 + 4	3 + 4
Wheat	25.00	20.00	36.00	36.00
Wheat (cracked)		0.20		
Barley	30.00	26.00	30.00	25.00
Barley (cracked)		0.20		
Rye bran		15.00		15.00
Wheat bran	9.00		9.00	
Corn	9.00	7.00		
Sugar beet pulp, molasses		2.50	2.00	2.00
Soybeans, toasted		2.00		
Soybean extraction meal	12.00	12.00	13.00	12.00
Raps extraction meal	9.00	10.00	1.00	1.00
Rapeseed oil	2.50	2.50		1.00
Linseed	0.80	0.70	1.40	1.40
Yeast	0.50			
Calcium carbonate	1.00	0.70	0.80	1.60
Calcium sodium phosphate	0.60	0.80	0.60	0.50
Sodium chloride	0.50	0.40	0.30	0.40
Monocalcium phosphate	0.10			
Baking and pastry industry (wafer flour)			5.90	4.10

All values have been rounded. Additives per kg: Nutritional additives: 12,000 IU vitamin A; 2000 IU vitamin D3; 120 mg vitamin E; 150 mg iron as ferrous sulfate monohydrate; 9 mg copper as copper(II) sulfate pentahydrate; 9 mg copper as dicopper chloride trihydroxide; 60 mg zinc as zinc oxide; 60 mg zinc as zinc chloride hydroxide monohydrate; 50 mg manganese as manganese(II) oxide; 50 mg manganese as glycine manganese chelate hydrate; 2 mg iodine as calcium iodate, anhydrous; 0.35 mg selenium as sodium selenite. Zootechnical additives: 500 FYT 6-phytase EC 3.1.3.26. Technological additives: Formic acid <E 236>; propionic acid <E 280>.

**Table 2:** Calculated nutritional values of the experimental and control diets for lactation sows.

Energy and Nutritional Content (g/kg as Fed)	Control 1 + 2	Experimental 1 + 2	Control 3 + 4	Experimental 3 + 4
Crude Protein	175	175	165	165
Crude Fat	57	57	40	40
Crude Fiber	50	50	55	55
Crude Ash	60	58	60	60
MJ ME/kg	13.4	13.4	13.2	13.2
Ca	8.5	8.5	8.5	8.5
P	6.5	6.5	5.3	5.3
Lys	10.5	10.5	10.0	10.0
Met	3.5	3.5	3.5	3.5

All values are overall target formulation values.

**Table 3:** Comparison of the energy and nutritional content of rye and rye bran.

Energy and Nutritional Content (g/kg DM)	Rye	Rye Bran
DM content [g/kg as fed]	891	897.5
Crude Ash [g/kg]	17.1	53.1
Crude Protein [g/kg]	92.5	166.6
Crude Fat [g/kg]	17.4	30.5
Crude Fiber [g/kg]	23.4	64.1
Starch [g/kg]	638.3	245.2
ME [MJ/kg as fed]	13.87	12.29
ME [MJ/kg DM]	15.60	13.7
Ca [g/kg]	<1.0	<1.0
P [g/kg]	2.9	10.9
Zea [µg/kg]	<10	14
DON [µg/kg]	<200	<200
Fructane [%]	2.91	6.15
Soluble Pentosane [%]	0.9	2.0–3.0
Total Pentosane [%]	9.19	13.0–15.0

Values have been rounded.

**Table 4:** Analyzed nutrient composition of compound feeds (g/kg DM).

Energy and Nutritional Content (g/kg DM)	Control	Experimental
DM content [g/kg as fed]	875.8 ± 8.1	881.8 ± 13.1
Crude Ash [g/kg]	53.7 ± 4.5	56.5 ± 11.7
Crude Protein [g/kg]	184.6 ± 21.7	185.0 ± 22.8
Crude Fat [g/kg]	47.3 ± 16.8	50.2 ± 17.2
Crude Fiber [g/kg]	48.7 ± 6.4	50.0 ± 7.3
Starch [g/kg]	457.0 ± 62.8	438.8 ± 61.0
ME [MJ/kg as fed]	13.1 ± 0.2	13.2 ± 0.1
ME [MJ/kg DM]	14.9 ± 0.1	14.9 ± 0.1
Calcium [g/kg]	8.1 ± 1.5	9.4 ± 3.3
Phosphorus [g/kg]	6.2 ± 0.6	6.1 ± 1.1
Zearalenone (ZEA) [µg/kg]	<10	<10
Deoxynivalenol (DON) [µg/kg]	<200	<200

**Table 5:** Fructane and pentosane contents of the respective raw materials and feeds.

	Wheat Bran	Rye Bran	Control	Experimental
Dry matter %	88.9 ± 0.0	90.1 ± 0.0	89.7 ± 1.6	89.3 ± 1.3
Fructans %DM	1.91 ± 0.01	4.78 ± 0.10	0.67 ± 0.16	0.84 ± 0.04
Total Pentosans %DM	18.95 ± 0.71	24.68 ± 0.78	10.79 ± 0.02	10.55 ± 0.21

**Table 6:** Performance parameters of sows in the control (C) and experimental group (E).

Parameter	Timepoint	Control	Experimental	*p*-Value
Bodyweight Sow (kg)	Housing	265.1 ± 33.3	259.0 ± 36.1	0.2615
	Weaning	235.1 ± 34.8	228.3 ± 38.5	0.2475
	Difference	−29.88 ± 12.15	−31.52 ± 12.64	0.3842
Backfat thickness (mm)	Housing	14.9 ± 2.92	14.2 ± 3.31	0.3303
	Weaning	13.0 ± 2.38	11.8 ± 2.58	**0.0303**
	Difference	−1.88 ± 1.54	−2.31 ± 2.06	0.2927

*p*-value < 0.05 was considered significant (in bold).

**Table 7:** Piglet weights (kg) of the control and experimental group at different timepoints.

n(C/E)	Day	Control	Experimental	*p*-Value
697/675	0	1.25 ± 0.34	1.20 ± 0.31	0.1291
684/656	1	1.33 ± 0.35	1.27 ± 0.33	0.0725
596/569	7	2.18 ± 0.56	2.16 ± 0.50	0.6703
563/562	14	3.72 ± 0.93	3.74 ± 0.86	0.6406
563/544	20	5.07 ± 1.24	5.10 ± 1.21	0.6767

*p*-value < 0.01 was considered significant.

**Table 8:** Reproduction parameters of the control and experimental sows.

Parameter	Control Diet (C)	Experimental Diet (E)	*p*-Value
Born alive	16.76 ± 3.14	17.00 ± 2.60	0.7236
Stillborn	1.00 ± 1.16	1.38 ± 1.55	0.2351
Mummified	0.32 ± 0.62	0.49 ± 1.07	0.3992
Weaned	13.29 ± 1.06	13.08 ± 1.23	0.4353

*p*-value < 0.05 was considered significant.

**Table 9:** Average estimated colostrum intake per suckling piglet (g).

Birthweight Category	n(C/E)	Control	Experimental	*p*-Value
Light (<1000 g)	170/176	203.0 ± 39.2	214.3 ± 35.9	**0.007**
Medium (1000–1500 g)	367/377	291.3 ± 39.0	298.5 ± 36.4	**0.010**
Heavy (>1500 g)	160/122	388.1 ± 48.4	381.0 ± 35.8	0.179

*p*-value < 0.05 was considered significant (in bold).

**Table 10:** Milk production per sow per day during lactation (calculated milk production divided by number of lactation days).

Estimated Average Daily Milk Production per Sow (kg)		
Control	Experimental	*p*-Value
11.36 ± 1.63	11.83 ± 1.41	0.1612

## Data Availability

The data presented in this study are available on request from the corresponding author.
